# Electron Paramagnetic Resonance Study on Oxygen Vacancies and Site Occupations in Mg-Doped BaTiO_3_ Ceramics

**DOI:** 10.3390/ma12091525

**Published:** 2019-05-09

**Authors:** Dayong Lu, Yongshun Zheng, Longfei Yuan

**Affiliations:** 1 Key Laboratory for Special Functional Materials in Jilin Provincial Universities, Jilin Institute of Chemical Technology, Jilin 132022, China; 13634327930@163.com (Y.Z.); yuanlf16@mails.jlu.edu.cn (L.Y.); 2 College of Chemistry, Jilin University, Changchun 130012, China

**Keywords:** barium titanate ceramics, dielectric properties, oxygen vacancies, site occupations, electron paramagnetic resonance

## Abstract

Nominal (Ba_1−*x*_Mg*_x_*)TiO_3_ (*x* = 0.015) (BM1T) and (Ba_1−*x*_Mg*_x_*)TiO_3_ (*x* = 0.03–0.20) (BMT) ceramics were prepared by the mixed-oxide route at sintering temperatures (*T*_s_) of 1200−1400 °C and 1200 °C, respectively. The solubility limit of Mg^2+^ in BMT was determined by XRD to be *x* = 0.05, and evidence was found for occupation of the A site by Mg^2+^. Electron paramagnetic resonance (EPR) was employed as a key technique to investigate the effect of *T*_s_ on oxygen vacancies in BM1T. The structure of BM1T changed from pseudocubic at *T*_s_ = 1200 °C to tetragonal at 1300 °C to mixed phases of hexagonal and tetragonal at 1400 °C. When *T*_s_ ≥ 1300 °C, a *g* = 1.956 EPR signal was observed at *T* = −188 °C and assigned as ionized oxygen vacancies. Mg^2+^ exhibited amphoteric behavior of substituting for the double cation sites. When *T*_s_ = 1400 °C, B-site Mg^2+^ and oxygen vacancies mainly existed in the hexagonal phase and A-site Mg^2+^ was dominant in the tetragonal phase. The higher tan *δ* was attributed to the higher concentrations of oxygen vacancies and Ti^3+^ in the hexagonal phase.

## 1. Introduction

BaTiO_3_-based ceramics are widely used in modern electronics because of their excellent ferroelectric and piezoelectric properties, and various dopants have been adopted to achieve high application performance. Singly doped magnesium (Mg^2+^) and Mg and rare earth co-doped BaTiO_3_ (ABO_3_) compounds that satisfy X7R or X8R specification have found applications in multilayer ceramic capacitors (MLCCs) [1,2,3,4,5,6].

Many transition metal ions with lower valence states occupy the B site when doped in BaTiO_3_, for example, Mn^2+^ [7,8]. Similar to these dopants, Mg^2+^ was also considered to be substituted for the B site as an acceptor because 6-CN Mg^2+^ is closer to Ti^4+^ in ionic size, and the defect notation was written as ${\mathrm{Mg}}_{\mathrm{Ti}}^{\prime \prime }$ according to the defect notation proposed by Kröger and Vink [9]. Considering the electroneutrality, ${\mathrm{Mg}}_{\mathrm{Ti}}^{\prime \prime }$ was usually compensated by one oxygen vacancy ($V_{O}^{••}$) and ${\mathrm{Mg}}_{\mathrm{Ti}}^{\prime \prime }-V_{O}^{••}$ pairs were supposed to exist in BaTiO_3_ [10,11,12,13].

At present, two scientific problems remain unsettled and need further investigation: (1) direct evidence for observing $V_{O}^{••}$ in Mg-doped BaTiO_3_ is still lacking; and (2) the possibility of occupying the A site for Mg^2+^ has not been determined. Our previous study confirmed the amphoteric behavior of Dy^3+^ in BaTiO_3_, which can occupy both A and B sites [14,15,16]. The ionic radius of 12-coordinate Dy^3+^ at the A site is 1.19 Å [14], which is little smaller than Mg^2+^ (1.23 Å) with the same coordinate number (CN). Ionic radii with different CN are given in Table 1 [17].

The amphoteric behavior of Dy^3+^ and the similar ionic radii between Dy^3+^ and Mg^2+^ indicate that although the structures and properties of B-site Mg-doped BaTiO_3_ have been studied, the possibility of Mg^2+^ occupying the A site should not be excluded. Although MgTiO_3_ has a distorted rhombohedral structure [18], which is completely different from the perovskite structure of BaTiO_3_, the difference in crystalline structure between MgTiO_3_ and BaTiO_3_ may not be a key factor for Mg^2+^ to enter the A site. Thus, the amphoteric nature of Mg^2+^ in BaTiO_3_ is still a scientific problem to be clarified.

In this work, BaTiO_3_−MgTiO_3_ (BMT) solid solutions were prepared at different sintering temperatures. At a lower sintering temperature (*T*_s_) such as 1150 °C, Mg^2+^ was considered to segregate to the surfaces of the grains and play an important role in the formation of the core–shell structure [6]. Therefore, a longer sintering time in this work was used to ensure the incorporation of Mg^2+^ into the BaTiO_3_ lattice. The site occupation and amphoteric behavior of Mg^2+^ and the dependence of $V_{O}^{••}$ on *T*_s_ were investigated. The electron paramagnetic resonance (EPR) technique was employed to detect the existence of $V_{O}^{••}$ in the low-temperature range.

## 2. Methods

Nominal (Ba_1−*x*_Mg*_x_*)TiO_3_ (*x* = 0.015) (BM1T) were prepared by the mixed-oxide method, described elsewhere [19], from reagent-grade BaCO_3_, MgO, and TiO_2_ powders. The molded pellets were sintered at 1200, 1300, and 1400 °C, respectively, for 12 h in air to form ceramics. In addition, (Ba_1−*x*_Mg*_x_*)TiO_3_ (*x* = 0.03, 0.05, 0.07, 0.10, 0.15, 0.20) (BMT) were prepared at 1200 °C for 12 h to investigate the occupation of Mg^2+^ at the A site.

Powder X-ray diffraction (XRD) data were collected using a DX-2700 X-ray diffractometer (Dandong Haoyuan, Dandong, China). The lattice parameters were calculated by MS Modeling (Accelrys, Inc., San Diego, CA, USA) using Rietveld refinement in the Reflex Package and Cu Kα1 radiation (λ = 1.540562 Å). Scanning electron microscope (SEM) investigations were performed using an EVOMA 10 SEM (Zeiss, Oberkochen, Germany) operated at 15 kV. The sample surfaces were first polished and then thermally etched at the same sintering temperatures for a few minutes before SEM measurement. The dielectric properties were investigated at 1 kHz, from −75 to 200 °C, at a heating rate of 2 °C/min using a Concept 41 dielectric/impedance spectrometer (Novocontrol) with an applied voltage of 1 V. Temperature-dependent electron paramagnetic resonance (EPR) measurements were performed using an A300-10/12 X-band spectrometer (Bruker, Rheinstetten, Germany) operating at 9.43 GHz. The EPR cavity of the spectrometer was replaced with an ER 4102ST cavity.

## 3. Results

**Figure 1:**
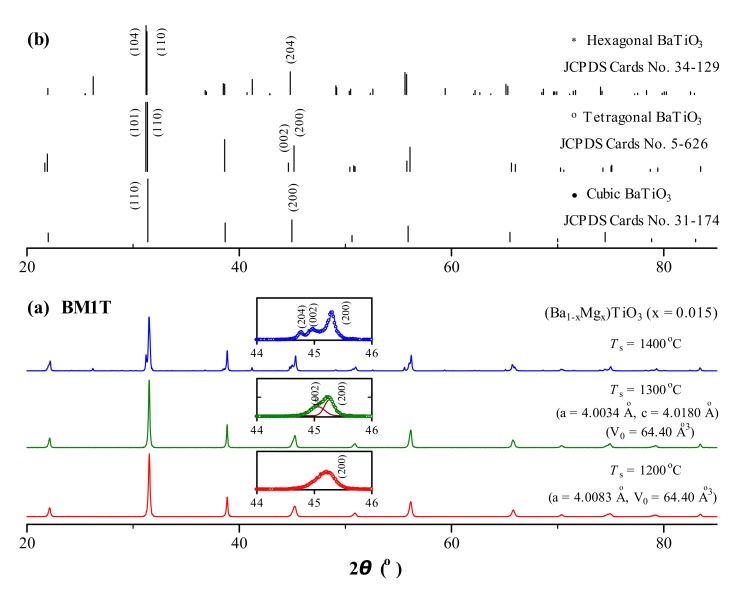
(**a**) Powder XRD patterns of (Ba_1−*x*_Mg*_x_*)TiO_3_ (*x* = 0.015) (BM1T) ceramics prepared at *T*_s_ = 1200–1400 °C. Insets show enlarged diffraction peaks in the vicinity of 45°. The lattice parameters are given. (**b**) Simulated XRD patterns of BaTiO_3_ with cubic, tetragonal, and hexagonal structures.

Powder XRD patterns of nominal (Ba_1−*x*_Mg*_x_*)TiO_3_ (*x* = 0.015) (BM1T) ceramics prepared at *T*_s_ = 1200–1400 °C are shown in Figure 1. BM1T sintered at *T*_s_ = 1200 °C exhibited a pseudocubic perovskite structure (space group: *Pm*3*m*) marked by a symmetric and broad characteristic (200) peak at ~45° (Figure 1a, inset). As *T*_s_ was increased to 1300 °C, this peak evolved into slight (002)/(200) splitting (Figure 1a, inset) and BM1T had a single-phase tetragonal structure (space group: *P*4*mm*), similar to the tetragonal BaTiO_3_ (JCPDS Cards No. 5–626) (Figure 1b). When *T*_s_ = 1400 °C, the peak at ~45° evolved into an overlapping of the tetragonal (002)/(200) peaks and the (204) peak (Figure 1a, inset) of the hexagonal BaTiO_3_ (space group: *P*63/*mmc*) (JCPDS Cards No. 34–129) (Figure 1b), i.e., the tetragonal and hexagonal phases coexisted in BM1T. It was inferred from the main (110) peak at ~31° that the amount of the hexagonal phase was approximately 30% of the tetragonal phase for BM1T sintered at *T*_s_ = 1400 °C.

**Figure 2:**
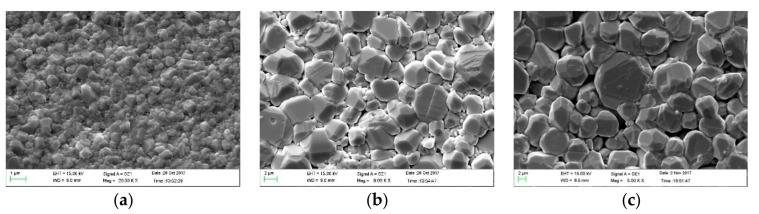
SEM images of polished and thermally etched surfaces of BM1T sintered at *T*_s_ = (**a**) 1200, (**b**) 1300, and (**c**) 1400 °C.

SEM images of BM1T are shown in Figure 2. BM1T exhibited an inhomogeneous grain size distribution and the grains rapidly grew from <1.0 to 10 μm with increasing *T*_s_.

**Figure 3:**
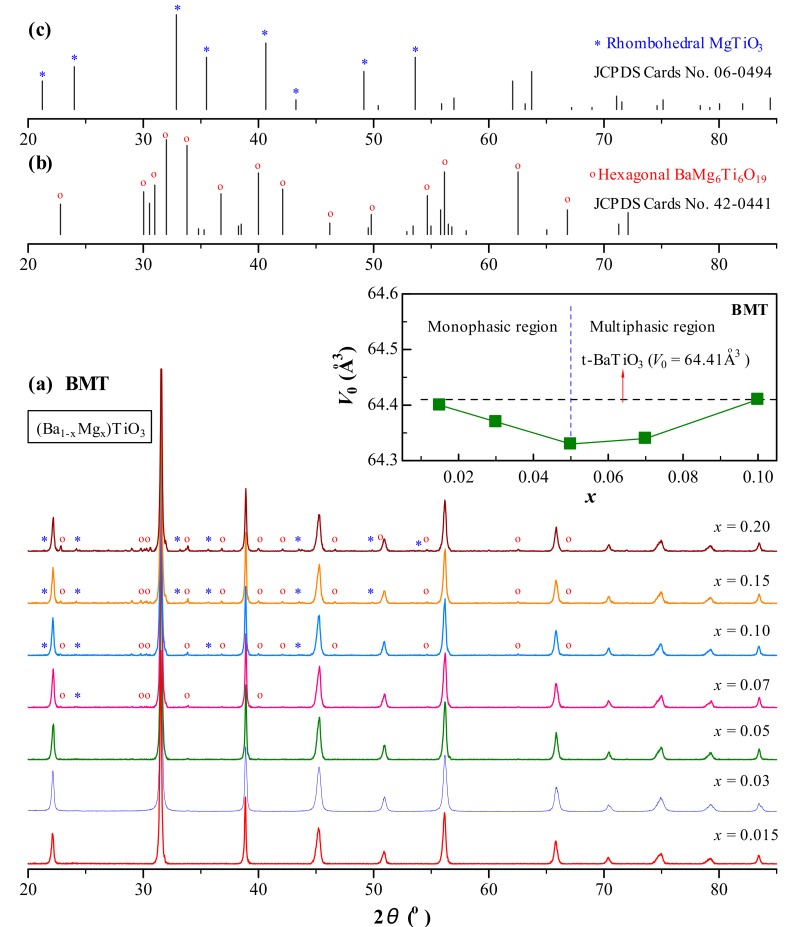
XRD patterns of (**a**) (Ba_1−*x*_Mg*_x_*)TiO_3_ (*x* = 0.03‒0.20) (BMT) ceramics sintered at *T*_s_ = 1200 °C. Inset depicts variation in *V*_0_ as a function of *x*. Simulated XRD patterns of (**b**) hexagonal BaMg_6_Ti_6_O_19_ (JCPDS Cards No. 42‒0441) and (**c**) rhombohedral MgTiO_3_ (JCPDS Cards No. 06‒0494).

XRD patterns of nominal (Ba_1−*x*_Mg*_x_*)TiO_3_ (*x* = 0.015‒0.20) (BMT) ceramics sintered at *T*_s_ = 1200 °C are shown in Figure 3. BMT had a pseudocubic perovskite structure up to *x* = 0.05. The secondary phases of the hexagonal BaMg_6_Ti_6_O_19_ [20] and the rhombohedral MgTiO_3_ appeared in BMT when *x* ≥ 0.07. Thus, the solubility limit of Mg^2+^ in BMT sintered at *T*_s_ = 1200 °C was determined by XRD to be *x* = 0.05. The variation in unit cell volume (*V*_0_) as a function of *x* for BMT is shown in the inset in Figure 3. In the monophasic region of *x* ≤ 0.05, *V*_0_ decreased linearly with increasing *x*. In the multiphasic region of *x* > 0.05, *V*_0_ increased.

**Figure 4:**
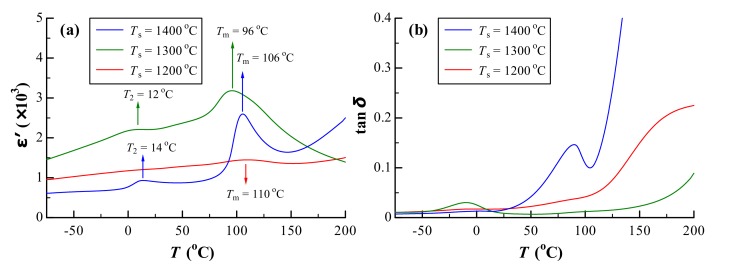
Temperature dependencies of (**a**) dielectric permittivity (*ε*’) and (**b**) dielectric loss (tan δ) for BM1T sintered at *T*_s_ = 1200, 1300, and 1400 °C.

Temperature dependencies of the dielectric permittivity (*ε*’) and dielectric loss (tan δ) for BM1T are shown in Figure 4. The *ε*’–*T* curve of BM1T sintered at *T*_s_ = 1200 °C was smooth and even, showing a rounded hill at around *T*_m_ = 110 °C. The Curie peak of BaTiO_3_ was dramatically suppressed due to Mg doping, and this ceramic satisfied the X8S specification (|(*ε*’−*ε*’_RT_)/*ε*’_RT_| ≤ 22% in a temperature range from −55 to 125 °C) with *ε*’_RT_ = 1200. BM1T exhibited a very low tan δ (0.0176) at room temperature and lower tan δ (<0.05) in a *T* range of −55 to 110 °C. Subsequently, tan δ increased with increasing *T*.

When *T*_s_ = 1300 °C, the *ε*’–*T* curve of BMT exhibited a bimodal structure, corresponding to a tetragonal–cubic (*t*–*c*) transition at dielectric peak temperature *T*_m_ = 96 °C and an orthorhombic–tetragonal (*o*–*t*) transition at *T*_2_ = 12 °C.

As *T*_s_ was increased to 1400 °C, the bimodal feature in the *ε*’–*T* curve became more distinct and *t*–*c* and *o*–*t* transitions occurred at *T*_m_ = 106 and 14 °C, respectively. The *ε*’_RT_ decreased and tan δ increased rapidly above *T* = 50 °C.

**Figure 5:**
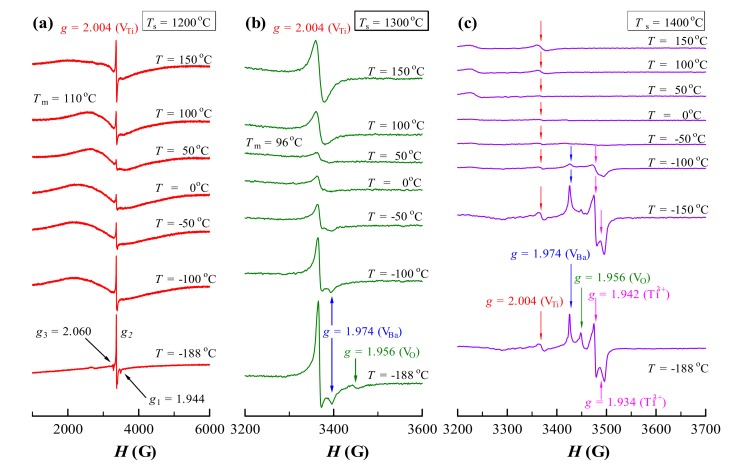
Temperature-dependent EPR spectra for BM1T sintered at *T*_s_ = (**a**) 1200, (**b**) 1300, and (**c**) 1400 °C.

Temperature-dependent EPR spectra for BM1T are shown in Figure 5. For BM1T sintered at *T*_s_ = 1200 °C, only the *g* = 2.004 signal existed over the measuring temperature (*T*) range of −188 to 150 °C (Figure 5a). This signal was assigned as ionized Ti vacancies [21,22,23]. The *g* = 2.004 signal was activated in the cubic phase above *T*_m_ and in the rhombohedral phase below *T* = −100 °C. This activation confirmed the nature of Ti vacancies [23]. The pair of weak lines denoted as *g*_1_ = 1.944 and *g*_3_ = 2.060 appeared at *T* = −188 °C, forming a centrosymmetric pattern around *g*_2_ = 2.004. This phenomenon is similar to the low-temperature EPR spectrum observed for (Ba_0.85_Sr_0.15_)TiO_3_ [24], which may relate to the occupation of Mg^2+^ on the A site.

When *T*_s_ = 1300 °C, except for the *g* = 2.004 signal, two additional signals at *g* = 1.974 and 1.956 observed at *T* = −188 °C (Figure 5b) were assigned as ionized Ba [14,22] and oxygen ($V_{O}^{••}+e\to V_{O}^{•}$) [19] vacancies, respectively.

BM1T sintered at *T*_s_ = 1400 °C existed in mixed forms of the hexagonal and tetragonal phases. Five EPR signals appeared below *T* = −100 °C and their intensity increased with decreasing *T* (Figure 5c). The presence of three signals at *g* = 2.004, 1.974, and 1.957 implies the coexistence of $V_{\mathrm{Ba}}^{\prime \prime }$, $V_{\mathrm{Ti}}^{\prime \prime \prime \prime }$, and $V_{O}^{••}$. We attributed two additional signals at *g* = 1.934 and 1.942 to a hexagonally distorted d^1^ ion from Ti^3+^ (${\mathrm{Ti}}_{\mathrm{Ti}}^{\prime }$) because low temperatures can effectively prolong the spin–lattice relaxation time (*τ*) [19,22]. This indicates that during high-temperature sintering of *T*_s_ = 1400 °C, the electrons in BM1T can be trapped by Ti^4+^ ions to cause a reduction from Ti^4+^ to Ti^3+^. It has been reported that the (Ba_1−*x*_Ca*_x_*)TiO_3_ (*x* = 0.03) ceramic sintered at *T*_s_ = 1500 °C showed a more ordered tetragonal structure, and only a Ti^3+^-related signal at *g* = 1.932 was observed at *T* = −188 °C [19,22]. However, this signal did not appear in the tetragonal BMT sintered at *T*_s_ = 1300 °C (Figure 5b). In the mixed hexagonal and tetragonal phases of BMT sintered at *T*_s_ = 1400 °C, the Ti^3+^-related signal split into two signals at *g* = 1.934 and 1.942. It is obvious that these two signals originated from the hexagonal phase in BM1T.

## 4. Discussion

### 4.1. Site Occupation of Mg^2+^ in BM1T at Different Sintering Temperatures

On the basis of a simple comparison of 12-CN ionic size between Ba^2+^ (1.61 Å) and Mg^2+^ (1.23 Å) and 6-CN ionic size between Ti^4+^ (0.605 Å) and Mg^2+^ (0.72 Å), a continuous decrease in *V*_0_ with *x* (≤0.05) for BM1T sintered at *T*_s_ = 1200 °C (Figure 3, inset) provides sufficient evidence for occupation of the A site by Mg^2+^. When *x* is higher than the solubility limit of 0.05, Mg^2+^ cannot continuously enter the A site, accompanied by separation of Mg-rich phases (Figure 3). The appearance of $V_{O}^{••}$ can be considered as an indication of the existence of Mg^2+^ at the B site, i.e., forming ${\mathrm{Mg}}_{\mathrm{Ti}}^{\prime \prime }-V_{O}^{••}$ pairs [10,11,12,13]. BO_6_ octahedrons are characteristic of the perovskite lattice. Hence, higher energy is required to incorporate doping ions into the B site. It is inferred that the sintering temperature of *T*_s_ = 1200 °C is too low to incorporate Mg^2+^ into the B site because the $V_{O}^{••}$-related EPR signal was not observed (Figure 5a). On the other hand, BM1T has a pseudocubic structure and its *V*_0_ (= 64.40 Å^3^) is equal to the tetragonal BaTiO_3_ (*V*_0_ = 64.41 Å^3^, JCPDS Card No. 6-526). This implies that Mg^2+^ tends to remain close to the surfaces of the grains and plays an important role in the temperature-stable X8S behavior in BM1T, as suggested by Chang et al. [5]. At this time, Mg^2+^ exists only at the A site as ${\mathrm{Mg}}_{\mathrm{Ba}}^{\times }$.

El Ghadraoui et al. indicated that the solubility limit of Mg^2+^ in (Ba_1−*x*_Mg*_x_*)TiO_3_ was 0.15. They neglected a small amount of the secondary phases of BaMg_6_Ti_6_O_19_ and MgTiO_3_, which also appeared in their samples with *x* ≥ 0.05 [25]. Their report undoubtedly supports that Mg^2+^ may exist at the A site.

When *T*_s_ was increased to 1300 °C, $V_{O}^{••}$ and $V_{\mathrm{Ba}}^{\prime \prime }$ were detected (Figure 5b), revealing that some Mg^2+^ ions transferred from the A site to the B site during the cooling process of ceramic sintering, accompanied by the creation of $V_{O}^{••}$. However, the numbers of ${\mathrm{Mg}}_{\mathrm{Ti}}^{\prime \prime }$ and $V_{O}^{••}$ were too small to induce the hexagonal phase.

When *T*_s_ = 1400 °C, more Mg^2+^ ions enter the B site. The concentration of ${\mathrm{Mg}}_{\mathrm{Ti}}^{\prime \prime }-V_{O}^{••}$ was high enough to cause phase splitting into hexagonal and tetragonal (Figure 5c). The hexagonal phase in BM1T originated from ${\mathrm{Mg}}_{\mathrm{Ti}}^{\prime \prime }-V_{O}^{••}$ defect complexes. Kirianov et al. and Dang et al. also reported a similar result on the mixed phases for Ba(Ti_1−_*_x_*Mn*_x_*)O_3_ with *x* < 0.03 [26,27]. The Jahn–Teller distortion encased by the ${\mathrm{Mn}}_{\mathrm{Ti}}^{\prime }$ ions is proposed to be the driving force of the phase transition from tetragonal to hexagonal [28]. This implies that ${\mathrm{Mg}}_{\mathrm{Ti}}^{\prime \prime }$ and ${\mathrm{Mn}}_{\mathrm{Ti}}^{\prime }$ acceptors on the Ti sites in BaTiO_3_ play the same role in the formation of the hexagonal phase. Thus, ${\mathrm{Mg}}_{\mathrm{Ti}}^{\prime \prime }$ and $V_{O}^{••}$ mainly exist in the hexagonal phase, and ${\mathrm{Mg}}_{\mathrm{Ba}}^{\times }$ is predominant in the tetragonal phase.

As a whole, Mg^2+^ ions in BM1T sintered at *T*_s_ ≥ 1300 °C exhibited amphoteric behavior, i.e., Mg^2+^ existed at the A site as ${\mathrm{Mg}}_{\mathrm{Ba}}^{\times }$ and at the B site as ${\mathrm{Mg}}_{\mathrm{Ti}}^{\prime \prime }$.

### 4.2. Oxygen Vacancies and Dielectric Loss

The $V_{O}^{••}$ can be detected by the EPR technique for Mg-doped BaTiO_3_. It is not easy to create $V_{O}^{••}$ when *T*_s_ is lower than 1200 °C and tan *δ* at *T*_s_ = 1300 °C is astonishingly low over the *T* range of −55 to 150 °C (tan *δ* ≤ 0.03).

The increase in *T*_s_ can create $V_{O}^{••}$ and ${\mathrm{Ti}}_{\mathrm{Ti}}^{\prime }$, giving rise to phase splitting into hexagonal and tetragonal at *T*_s_ = 1400 °C. The high value of tan δ is attributed to the high concentrations of $V_{O}^{••}$ and ${\mathrm{Ti}}_{\mathrm{Ti}}^{\prime }$ in the hexagonal phase in BM1T (Figure 4).

## 5. Conclusions

The solubility of Mg^2+^ in (Ba_1−*x*_Mg*_x_*)TiO_3_ ceramics sintered at 1200 °C was 0.05, and rhombohedral MgTiO_3_ and hexagonal BaMg_6_Ti_6_O_19_ phases were observed with higher doping content. The evolution of unit cell volume provided sufficient evidence for the A-site occupation of Mg^2+^. The *x* = 0.015 ceramic had a pseudocubic crystal structure when the sintered temperature was 1200 °C and exhibited a temperature-stable X8S dielectric specification with *ε*′_RT_ = 1200. The structure transformed into a tetragonal phase when sintered at 1300 °C, and tetragonal and hexagonal phases coexisted when sintered at 1400 °C.

For *x* = 0.015 sintered above 1300 °C, the *g* = 1.956 signal observed at *T* = −188 °C was assigned as ionized oxygen vacancies ($V_{O}^{••}$). Mg^2+^ acted as an amphoteric doping ion with ${\mathrm{Mg}}_{\mathrm{Ba}}^{\times }$ and ${\mathrm{Mg}}_{\mathrm{Ti}}^{\prime \prime }$. ${\mathrm{Mg}}_{\mathrm{Ti}}^{\prime \prime }$ and $V_{O}^{••}$ mainly existed in the hexagonal phase and ${\mathrm{Mg}}_{\mathrm{Ba}}^{\times }$ was predominant in the tetragonal phase. Two EPR signals at *g* = 1.934 and 1.942 originated from the hexagonal phase in *x* = 0.015 and were related to Ti^3+^ which, along with $V_{O}^{••}$, is mainly responsible for the higher tan δ value.

## Figures and Tables

**Table 1:** Ionic radius as a function of coordinate number (CN).

Ion	CN	*r* (Å)
Ba^2+^	12	1.61
Ti^4+^	6	0.605
Ti^3+^	6	0.67
Mg^2+^	12	1.23
Mg^2+^	6	0.72
